# Cost and cost-effectiveness of attractive targeted sugar baits (ATSB) in the context of a phase III cluster randomized control trial in Western Province, Zambia

**DOI:** 10.1186/s12936-025-05716-9

**Published:** 2025-12-19

**Authors:** Brooke Mancuso, Erica Orange, Thomas P. Eisele, Ruth A. Ashton, Megan Littrell, Mulenga Matches, John M. Miller, Javan Chanda, Sosenna Assefa, Joseph Wagman, Kafula Silumbe, Busiku Hamainza, Kochelani Saili, Laurence Slutsker, Joshua Yukich

**Affiliations:** 1 Centre for Applied Malaria Research and Evaluation, Tulane School of Public Health and Tropical Medicine, New Orleans, LA USA; 2 PATH, Seattle, WA USA; 3 PATH, Washington D.C., WA USA; 4 PATH, Kaoma, Zambia; 5 PATH, Lusaka, Zambia; 6 National Malaria Elimination Centre, Lusaka, Zambia; 7 Atlanta, GA USA

**Keywords:** Cost, Cost-effectiveness, ICER, DALY, Malaria, ATSB, Clinical trial, Cluster randomized trial

## Abstract

**Background:**

Vector control is the most important malaria prevention strategy in Zambia. Attractive Targeted Sugar Baits (ATSB) are a potential new tool for vector control in this setting, which, if efficacious, would be intended to supplement insecticide-treated bed nets (ITNs) and indoor residual spraying (IRS). ATSBs target and kill sugar feeding mosquitoes, potentially limiting the spread of malaria. No information on the cost or cost-effectiveness of deployment of ATSB stations is currently available.

**Methods:**

A cluster randomized control trial (cRCT) was carried out in Western Province, Zambia to assess the efficacy of Sarabi v.1.2 ATSB stations in a highly malarious setting. Costs associated with the procurement, distribution, maintenance, and disposal of the ATSB stations were collected over a two-year period. These costs were assessed alongside the main trial efficacy outcomes to determine cost-effectiveness and potential budget impact on the deployment of ATSB stations in this setting. Total costs, incremental costs, incremental cost-effectiveness ratios (ICER) and budget impact were estimated using trial data. One-way, scenario and probabilistic sensitivity analysis were performed to further determine the impact of assumptions and uncertainty on cost-effectiveness estimates, and the potential cost implications of alternative deployment scenarios. Sub-group analysis was performed to determine the impact of deployment in settings with the most favorable effect scenarios.

**Results:**

The total cost of the intervention in the context of the cRCT was USD 1,261,515. ATSB cost accounted for 46% of the total cost followed by personnel (25%), supplies and transport (13% each), equipment (2%) and storage (1%). Over the two year (14-month intervention) this resulted in an estimated ICER of USD 79 per malaria incident case averted or USD 919 per disability-adjusted life year (DALY) averted and USD 10.08 per person-year protected. In a subset of high-density ATSB clusters ICER was USD 42 per incident case averted and USD 493 per DALY averted and USD 4.35 per person-year protected. Probabilistic sensitivity analysis indicated that deployment in areas with higher structure density may be more cost-effective, especially if potential cost-savings are considered. However, effect estimates in this subgroup were highly uncertain and not statistically significant. While the scenario appeared more cost-effective than the base case on the cost-effectiveness acceptability curve (CEAC), the probability of cost-effectiveness reached only around 70%, falling short of the commonly used 80% threshold and remaining relatively weak.

**Conclusions:**

ATSB Sarabi v.1.2 as deployed in western Zambia were not likely to be cost -effective. ATSB would need to demonstrate higher or more certain efficacy along with affordable alternative distribution strategies prior to any deployment at scale.

*Trial registration* The trial is registered on clinicaltrials.gov under registration number: NCT04800055.

**Supplementary Information:**

The online version contains supplementary material available at 10.1186/s12936-025-05716-9.

## Background

Malaria remains a major global health problem, with nearly 263 million cases and about 597,000 deaths worldwide in 2023, the majority of which occurred in sub-Saharan Africa [1]. Thanks to innovations in vector control and treatment, major reductions in the global malaria burden have been made over the last twenty years. However, despite these significant gains, progress has stalled, and new tools are needed to continue to reduce malaria burden. Increasing insecticide resistance and altered mosquito feeding behaviour necessitates innovative tools for malaria prevention [2–4]

Zambia remains a highly endemic country for malaria despite localized reductions in burden in recent years [5, 6]. The rainy season occurs between November and March each year and malaria infection peaks immediately following [5, 6]. Standard vector control, defined by the National Malaria Elimination Centre, is one ITN per two people in each household or IRS in the household within the last twelve months [6]. Details of the study site in Western Province can be found in Arnzen et al*.* [7].

Attractive Targeted Sugar Baits (ATSB) are a potential new approach to malaria vector control to address current challenges including insecticide resistance, and residual transmission (persistence of malaria parasite transmission despite high coverage of core indoor vector control measures) arising due to vector behaviour [8] ATSB utilize sugar as well as insecticides which are intended to function under an ‘attract-and-kill’ paradigm, targeting mosquitoes during facultative feeding [8, 9]. Previous studies and an entomological cluster trial in Mali have shown that ATSB deployment can lead to decreases in vector density and longevity, as well as sporozoite rate and entomological inoculation rate [8–11].

Current vector control tools, ITN and IRS, are generally considered to be highly cost-effective malaria prevention tools [12–15]. These current strategies are relatively inexpensive to distribute or apply, require limited behaviour change by users, provide long-lasting protection (months to years) and are highly effective at reducing exposure of large numbers of individuals to infectious bites from malaria transmitting mosquitoes owing to both individual bite prevention as well as community level effects on mosquito populations when deployed community wide [12–15].

Currently there is no evidence about the cost-effectiveness of ATSB using any product or distribution strategy. Cost and cost-effectiveness can be an important component of decision making around deployment of vector control tools, especially where combination strategies are considered [16, 17]. As such, it is critical to develop an evidence base on the cost and cost-effectiveness of various ATSB products and deployment strategies.

Three large-scale cluster randomized control trials (cRCT) of ATSB Sarabi v1.2 plus standard vector control vs. standard vector control alone were conducted in Zambia, Kenya and Mali to assess the efficacy of Sarabi v1.2 ATSB station on the prevention of clinical incidence of malaria [18]. This study examines the cost and cost-effectiveness of the deployment of ATSB Sarabi v 1.2 in the Zambia cRCT. The economic evaluation conducted in this paper follows the standardized guidelines outlined in the Consolidated Health Economic Evaluation Reporting Standards 2022 (CHEERS 2022) [19]. This approach aims to generate consistent outcomes to support decision-makers in programme planning and resource allocation [19].

## Methods

### Efficacy trial design

This study was conducted as part of a Phase III cRCT in Western Province, Zambia between November 2021 through June 2023 [18, 20]. Further details of the trial design are available in Attractive Targeted Sugar Bait Phase III Trial Group, 2022 including details of the ATSB intervention implementation [18, 20]. In brief, seventy clusters were assigned randomly (1:1) to an intervention arm, who received ATSB Sarabi v1.2 stations in the context of the standard of care of high vector control coverage (ITN and IRS), or to a control arm where clusters did not receive ATSB stations but did continue to have the standard of care high vector control coverage [20]. ATSB stations were deployed over a period of two calendar years (during the malaria transmission seasons only) and the primary outcome was the incidence of clinical malaria among a cohort of children from the study communities from ages 1–14 years. Malaria clinical incidence was measured by an assessment for fever or history of fever and histidine rich protein-2 rapid diagnostic test (RDT: SD Bioline Malaria Ag *P.f.*, Standard Diagnostics, South Korea, and Abbott Bioline Malaria Ag *P.f.*, Abbott, USA) at each monthly follow-up over the course of the six-month follow up period during the main transmission season of each year [18, 20] (see Table 1).

**Table 1:** Base case scenario assumptions

Parameter	Base case assumption
Efficacy estimate	9%
Discount rate	3%
Case fatality rate	0.002
DALY disability rate	0.02
Years life lost per fatality	33
Cost per ATSB	4.19
ATSB per eligible structure	2
Effectiveness population	All ages

### Study site and population

The Western Province trial site has one of the highest burdens of malaria in Zambia, where the estimated malaria incidence was 785 per 1000 people in 2021 [5]. Prevalence of children testing positive by rapid diagnostic test (RDT) was 47.4% during the high transmission season in the same year and *Anopheles funestus s.s.* is the dominant vector [18, 21]. The trial was conducted in three districts, Kaoma, Nkeyema and Luampa and is described in detail elsewhere [9–11]. A total of 246,785 people were estimated to live in these districts according to 2022 census data, while in the 70 trial clusters there was an estimated total population of 122,023 determined by baseline enumeration [7]. The 35 intervention clusters had a population of 61,505 at the time of the baseline enumeration (Table 2) [7]. The average household size in these communities was estimated to be 4.7 people per household and all areas were considered rural. Further information on the trial site and population can be found in Arnzen et al*.* [7]. A detailed narrative description of the intervention was developed through a review of project documents and interviews with key project implementation staff. The narrative was compiled and refined with iterative input from project staff. The final narrative was used to identify the activities and inputs/line items for which price and quantity or other cost related data was subsequently collected.

**Table 2:** ATSB intervention measures and outcomes, Zambia

	Year 1	Year 2	Total intervention period
ATSB shipped to Zambia	79,200	68,000	147,200
ATSB deployed	67,945	69,494	137,439
ATSB remaining end of intervention	–	–	9751
Households protected	11,638	12,045^**ǂ**^	–
Persons protected	61,505	63,658^**ǂ**^	–
Person-years	61,505	63,658	125,163
Structures covered	19,034	19,700^**ǂ**^	–
Kilos incinerated	13,940	14,864	28,804
Cases averted	7976	8256	16,232
DALY averted	710	686	1396
Malaria related deaths	1	3	4

^ǂ^Numbers calculated using baseline enumeration figures and adjusted for assumed growth rate of 3.5%

### Cost data collection

An ingredients approach was used to estimate the value of all resources used to deliver the intervention. Data on inputs, quantities and prices were collected using project administrative, financial and operational records, key informant interviews, receipts and invoices. Each input was assigned to a specific line-item category (ATSB, personnel, transport, supplies or equipment), a specific activity, a study year, and where possible to a specific study cluster. Inputs were also classified as capital or recurrent costs. For capital costs, an appropriate use life was identified using a combination of project records, key informant interviews or existing literature. Prices were collected in either Zambian Kwacha (ZMW) or United States Dollars (USD) and adjusted to 2023 USD equivalents. Research costs, such as rapid diagnostic tests for incidence measurement in the cohort, were excluded from consideration in this analysis. Resources that were considered for analysis included the above categories for only the ATSB distribution. Costs were defined as either recurrent, such as airtime or daily stipends, or capital, for goods which last longer than one year such as owned motor vehicles.

### Cost data analysis

The economic costs of ATSB deployment, monitoring, collection, and disposal were estimated using the provider perspective. The provider in this case includes the Zambian NMEC, healthcare workers, the non-governmental organization PATH: The Program for Appropriate Technology in Health (PATH), and any other relevant entity involved in the distribution, maintenance, and disposal of the ATSB. Costs included all direct costs for the procurement, distribution, maintenance, storage and disposal of ATSB in the thirty-five intervention clusters receiving ATSB from November 2021 through June 2023, covering two malaria transmission seasons. Neither costs associated with other vector control interventions, such as LLIN nor IRS, nor cost savings from reduced treatment were considered in base case scenario analysis. Costs were collected at the time incurred, and were converted to USD of the same year using the exchange rates from the World Currency Exchange rates and Currency. These costs were then inflated from the time of incurrence to June 2023 equivalents using the US Bureau of Labor and Statistics Consumer Price Index inflation rate. Any costs that were in ZMW were then converted to USD using the exchange rates from the World Currency Exchange. Finally, all costs were converted to 2023 Present Value using the assumed discount rate of three percent [22]. Capital costs were annuitized over the specific product’s expected lifetime using a discount rate of three percent in base case scenario analysis and valued based on the estimated amount of use time for each product during the course of the intervention.

Costs associated with the intervention were calculated for each study year. Total costs consisted of the sum of all costs across all intervention areas across both study years. Total costs were divided by relevant outcomes to produce estimates of cost per ATSB deployed, cost per household-year, cost per structure-year and cost per person-year.

#### Outcomes

Several outcomes were assessed in terms of resource use, these include the total number of ATSB stations sent out for deployment in the trial, the total number of persons and person-years protected in the intervention area, as well as the numbers of persons, structures and households in the intervention areas.

In terms of efficacy, cases averted were calculated based on incidence rate ratio estimated from the Intent-to-Treat (ITT) analysis of the (non statistically significant IRR of 0.91 or 9% reduction in cases) primary trial outcome and the average incidence rate in the control arm, these rates were then scaled to all-ages in the intervention population. Since the trial calculated incidence only among those 1 to 15 years of age, cases averted were calculated for the entire population assuming that the incidence rate ratios and incidence rate were the same in the entire population as they were in the cohort. This assumption was examined in sensitivity analysis.

Disability adjusted life years (DALYs) averted were calculated assuming an average lifespan of 72 years and estimating the years of life lived with disability (YLD) and the years of life lost (YLL) for the population of the intervention area. As mortality is the driving factor in DALYs for malaria and was not measured in the trial, an assumption for case fatality rate (CFR) was necessary to estimate YLL. CFR was assumed to be 0.002 per incident malaria case in children, an assumption taken from the lower bound confidence interval for a mild, acute case of an infectious disease [23]. Each child death was assumed to lead to 33 YLL(23, 24). A disability weight of 0.02 was assumed to calculate the years of life lived with disability(23). Averted YLD and averted YLL were summed to give the total DALYs averted [24].

### Cost model

Costs for standard of care vector control, whether in the study arm or intervention arm were considered equivalent for the purposes of this analysis and not measured. Where costs could not be directly assigned to a specific study cluster, either because they were shared across clusters or data on effort or use for allocation to cluster level was not available, these were assumed to be allocated evenly across clusters. Where the intervention description indicated that effort or cost varied for a line item and activity between clusters, shared costs were allocated to clusters based on the numbers of ATSB sent for deployment or ATSB monitor working days.

### Effect model

Thirty-five individuals per cluster between 12 months and 15 years of age were sampled for inclusion into the cohort during each year of the trial for assessment of the primary outcome [25]. Cumulative malaria case incidence for each arm was defined as the total number of clinical malaria cases (history of fever within the previous 48 h or a measured temperature of ≥ 37.5⁰C, plus a positive malaria rapid diagnostic test (RDT)) assessed using bi-weekly active case detection in the cohort [25]. A general linear model (GLM) with Poisson likelihood and log link function with random effects for cluster was used to estimate the incidence rates ratio for the intervention arm relative to the control group [26].

#### Base case scenario

A 9% efficacy rate from the main trial paper [25] was used as the base case efficacy for ATSB rollout. However, it should be noted that this result was non-significant. The assumptions made for the trial cost effectiveness were a 3% discount rate, an ATSB station cost of USD 4.19 per unit, and that the incident rate ratio and baseline incidence rate applied to the entire study population, not just those under the age of fifteen. Additional analysis was done to evaluate the cost-effectiveness when effect on malaria clinical case incidence was applicable only to the population under the age of 15.

#### One-way analyses

One-way and scenario analyses were conducted to assess the impact of the model and parameter assumptions on the conclusions of the base case scenario analysis. One-way analyses included number of ATSB per eligible structure, frequency of monitoring, discount rate, assumption that IRR applies to only those under the age of fifteen, price of ATSB, and cost savings from cases averted. Scenario analyses were also conducted to include combinations of changes to parameters and described more thoroughly below.

#### Probabilistic sensitivity analysis (PSA)

A probabilistic sensitivity analysis was performed to determine the variability surrounding the ICER estimates. A two-stage bootstrap was used to resample clusters and individuals in the cohort from the trial to capture variation in both the costs and the effects of the intervention due to the clustered trial design. Resampling of the control and intervention arm clusters were stratified for the bootstrap estimation. In short, the original effectiveness dataset was stratified into control and intervention arms, in each iteration a resample of 35 clusters (with replacement) was drawn for each study arm. Results of the cluster level cost estimates were attached for each cluster which was resampled in each iteration. Within each resampled cluster, a resample of individuals was taken (identical in size to the original cluster sample) with replacement. This new dataset was then used to re-estimate the main ITT regression model as well as calculate a new total cost of the trial. Ten-thousand iterations of this process were conducted to establish a data set of estimated total costs and effects (following the above methods for calculating cases, deaths and DALYs averted) which could then be further analysed.

The same process was applied to a subset of clusters characterized by high-density housing and, consequently, higher ATSB coverage. This subset was selected based on findings from the main trial, which suggested the possibility of increased intervention efficacy in such areas [25]. It is important to note that these efficacy results were also not statistically significant.

All ICER estimates (in terms of DALYs averted) were compared against a willingness to pay threshold of one- to three-times the Zambian GDP per capita, USD 1,457 and USD 4,371, respectively, through the construction of cost-effectiveness acceptability curves [27, 28]. While this paper uses 1 and 3 × GDP as willingness-to-pay thresholds, other methods and lower thresholds have also been suggested [29]. These would result in thresholds that are likely lower than 1 × GDP and always lower than 3xGDP [30].

#### Sub-group analysis

A sub-group analysis of the trial found evidence for a larger effect size in clusters where the density of structures was greater than 1 per hectare. There were 24 clusters that fit this definition, 14 intervention clusters and 10 control clusters. PSA was repeated for this subgroup of clusters only as described in the above section on PSA [25].

#### Cost-savings analysis

Net costs were also calculated assuming that cost-savings could be realized in proportion to the number of cases averted due to fewer antimalarial treatments being required from the health care provider. The value of these cost savings was estimated based on the cost of treating an uncomplicated malaria case (8.83 USD (2023)), converted from 2011 figures using the Consumer Price Index Inflation Calculator from the Bureau of Labor and Statistics ([15, 30]. A more recent study cited this figure as 7.84 USD (2023) per uncomplicated malaria case, but the slightly higher figure was used in the analysis [31]. However, the higher estimate was used in the analysis to present a best-case scenario. Approximately 60% of persons with an uncomplicated malaria case seek treatment overall in Zambia, though the proportion in Western Province Zambia is much lower (approximately 21.8%) [32]. Estimates of cost savings were examined using both values in sensitivity analyses [32].

## Results

### Outcomes

The ATSB intervention included installation, routine monitoring, and end-of-season removal of Sarabi v1.2 stations across 35 intervention clusters. Each eligible structure received two ATSB stations during deployment in each year, supported by community sensitization activities and a group of ATSB Monitors and Community Health Workers. Monitors were required to check every ATSB station at least once every two months and replace any damaged or missing stations, resulting in approximately one-third of all deployed ATSBs being issued during the monitoring period.

During the first deployment period of November 2021-June 2022 a total of 67,945 ATSBs were sent out for distribution, 40,862 during initial hang-up and 27,083 during the monitoring period (either to replace damaged or missing ATSB or for new installations).The second intervention year saw a similar deployment of ATSB stations with a total of 69,494 ATSB stations distributed (42,989 ATSB during deployment and 26,505 ATSB during monitoring activities). A total of 61,505 living within the intervention cluster boundaries, including buffer zones were considered protected in year one and after assuming and adjusting for a 3.5% population growth, this number was assumed to be 63,658 people in year two. Growth was assumed to be the same for houses and structures and therefore, based on enumeration data collected at baseline there were 11,609 households and 19,034 structures in the same area in year one. Growth rate was assumed to be approximately 3.5% for structures and households in year 2, as well. Calculating this from baseline enumeration rates, 12,045 households and 19,700 structures were in the intervention cluster boundaries in year two. A total of 17,856 kg of waste were incinerated over the two-year period, 3,002 in the first year and 14,863 kg in the second. Year two waste disposal included a large portion of year one hang-down ATSB stations and other materials (Table 2).

### Costs

**Fig. 1:**
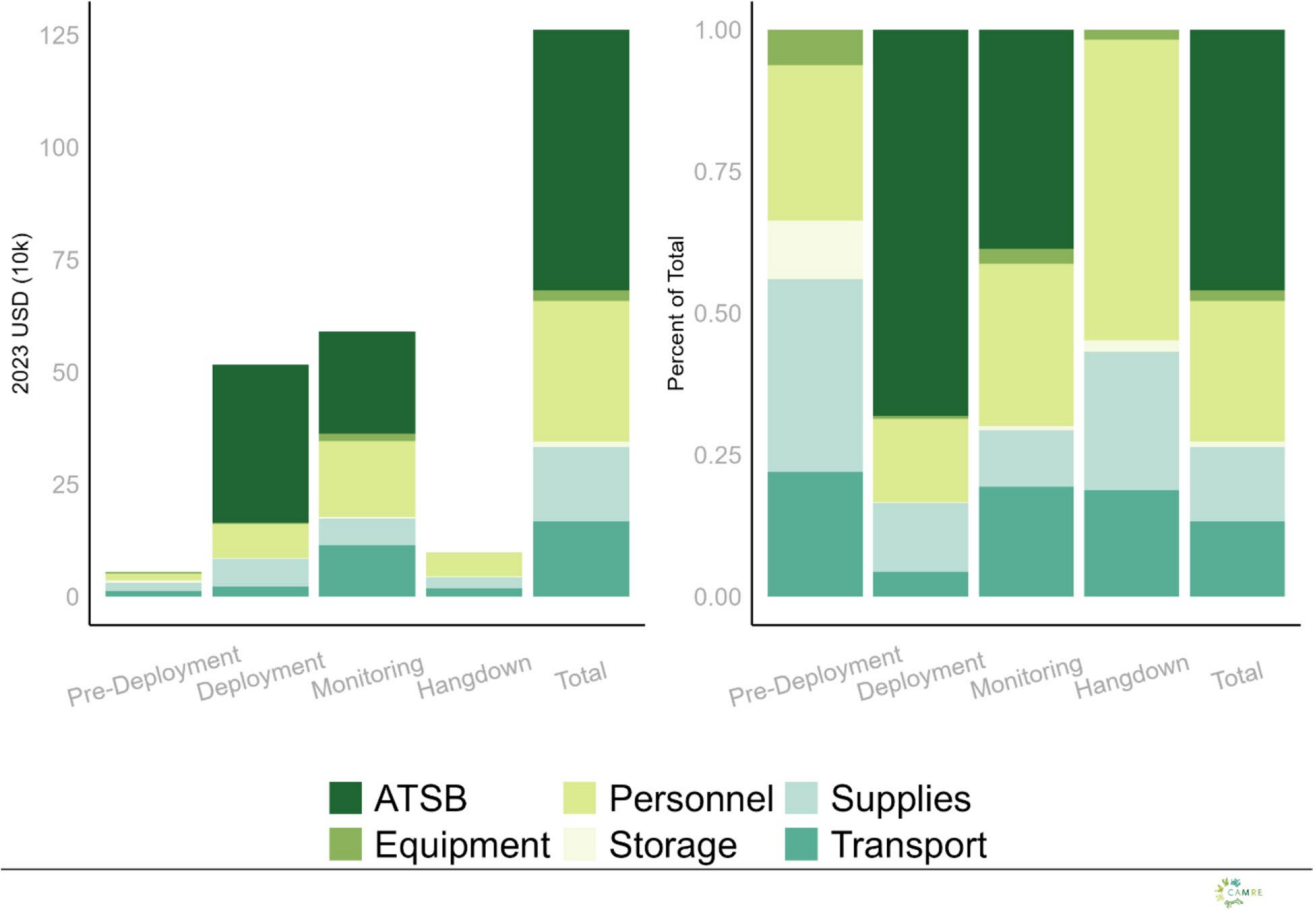
Total and proportional costs of ATSB by intervention phase and line-item category

The total cost of the intervention in year one and year two using base case assumptions was USD 630,242 and USD 631,273, respectively (Table 3). The total cost for having stations in place over the 14 months of intervention was therefore estimated to be USD 1,216,515 (Table 3). ATSB stations represented the largest share of total costs in the intervention under the base case scenario at USD 560,500 or 46% of the total costs, followed by personnel (25%), transport (13%) and supplies (13%), equipment (2%), and storage (1%). The distribution of ATSB intervention costs across clusters in 2023 USD for the base case scenario, presented as cost per ATSB, per household (HH), per person, and per structure can be seen in supplemental REF _Ref209387336 \h \* MERGEFORMAT figures 4 and REF _Ref209388129 \h \* MERGEFORMAT 5. Costs per household exhibited the greatest variation across clusters, while costs per ATSB were the most consistent. All costs were valued at their time of use, the majority of which were allocated to the installation and monitoring periods. Thus, installation and monitoring were the highest cost periods in the base case scenario ( REF _Ref185515102 \* MERGEFORMAT Fig. 1).

**Table 3:** Cost results from baseline case scenario, 2023 USD

Intervention Period	Year 1	Year 2	Total cost
Pre-deployment	25,121	30,432	55,553
deployment	258,927	257,298	516,225
Monitoring	302,722	287,697	590,419
Hang-down	43,472	55,846	99,318
All	630,242	631,273	1,261,515

After ATSB stations, personnel constituted the next largest line item in the base case scenario. and represented more than 50% of all the non-ATSB station costs (Fig. 1). Cost per ATSB station deployed was USD 9.11, cost per person-year (season) USD 10.08, cost per household-year (season) USD 53.40, and cost per structure-year (season) USD 32.57(Table 4).

**Table 4:** Incremental cost effectiveness for ATSB in 2023 USD, with subset analyses

	All clusters	High density subset clusters
Per case Averted	79.08	42.36
Per DALY	919.60	492.54
Per ATSB	9.11	7.91
Per household-year	53.40	22.64
Per person-year	10.08	4.35
Per structure-year	32.57	15.45

The cost per case-averted and cost per disability adjusted life year (DALY) averted—the incremental cost-effectiveness ratios—were estimated under base case scenario assumptions to be USD 79 per case-averted and USD 919 per DALY averted (Table 4).

### Sensitivity analysis

#### One-way sensitivity analyses

A one-way sensitivity examined cost and cost effectiveness under the assumption that ATSB were donated (i.e. priced at zero) to reflect a potential future scenario in which the Ministry of Health would not bear product cost. Under this analysis the total cost of the intervention was USD 342,060 in year one and USD 339,847 in year two in total USD 681,907(Table 5). Setting the price of ATSB stations to zero, cost per case averted fell to USD 43 (Table 5) and cost per DALY averted to USD 488. In the high-density cluster subset ICERs were estimated to be USD 42 per case averted and USD 493 per DALY averted (Table 4).

**Table 5:** Results of one-way sensitivity analyses and scenario analyses, Costs in 2023 USD

Parameter or scenario	Base case scenario value	Range	Justification	Cost USD	USD per case averted
Efficacy	9%	9–30%	Updates to ATSB may result in higher efficacy in the future	1,261,515	79–24
Cost per ATSB	4.19	0,2,10	Updates in design may cause variation	681,907–958,569–2,065,219	43–60–129
ATSB per eligible structure	2	2–6	Further study may change recommendation	1,261,515–2,420,731	79–151
Monitoring frequency	1 × Every other Month	0–2 × per month	Future programmatic strategies likely to include varied monitoring	671,095–1,851,934	*
Discount rate	3%	0–8%	Prevailing interest rates may change	1,249,933–1,281,922	79–80
Effectiveness demographic	All ages	Under 15 years only	Effect measured only in children 1–15 in study, uncertain if effects would carry to whole population	1,261,515	189

This table presents results from one-way sensitivity analyses and scenario testing for the cost-effectiveness of ATSB deployment

The base case reflects observed trial parameters, while alternative values were selected based on plausible programmatic or market-based changes. Each row presents the parameter varied, the base case value, the range used for analysis, and the justification for that range

Outcomes include the total cost of the intervention and the estimated cost per malaria case averted

Scenarios explore uncertainty in ATSB efficacy, unit cost, deployment density, monitoring frequency, discount rates, and the population group to which effectiveness is applied

*Asterisk indicates that case averted values are not directly comparable due to inconsistent denominators across scenarios

Additional one-way sensitivity analyses were done examining the number of ATSB per eligible structure, monitoring frequency, discount rate and the effectiveness demographic. Sensitivity analyses also explored a range of efficacy values, from the observed 9% reduction (IRR = 0.91; 95% CI 0.72–1.15, *p* = 0.42) in incidence to a 30% reduction, which reflects the effect size the trial was originally powered to detect [26].

#### Threshold analysis

**Fig. 2:**
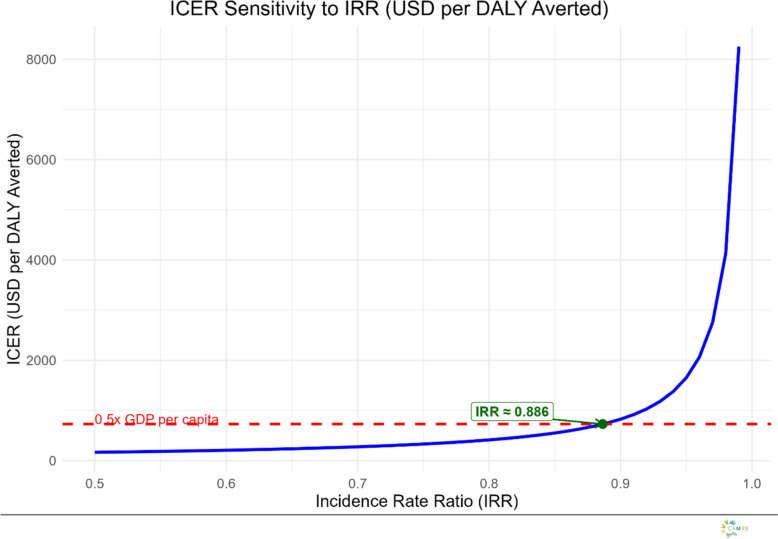
ICER Sensitivity to IRR (in USD per DALY averted): This curve illustrates the relationship between the incidence rate ratio (IRR) and the incremental cost-effectiveness ratio (ICER), expressed in USD per DALY averted. The analysis shows how the ICER increases as the IRR approaches 1, indicating reduced intervention effectiveness. The red dashed line represents the cost-effectiveness threshold of 0.5 × GDP per capita (USD 729). Values below this line are considered cost-effective under this benchmark under certain standards [42]

**Fig. 3:**
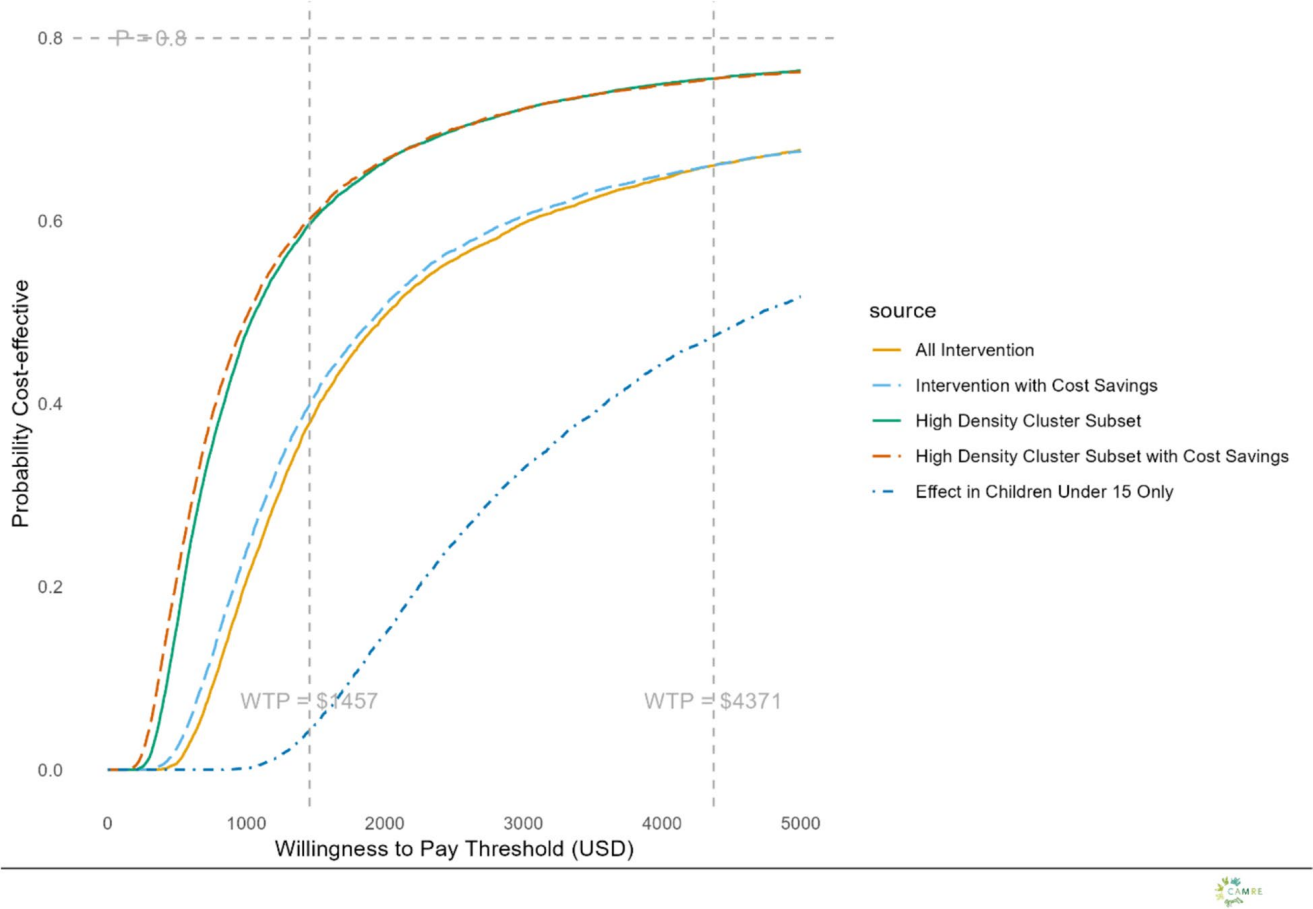
Cost-effectiveness acceptability curves: Each curve is based on 10,000 probabilistic iterations, reflecting uncertainty in both costs and effects. The curves show the probability that each scenario is cost-effective across a range of willingness-to-pay thresholds per DALY

**Fig. 4:**
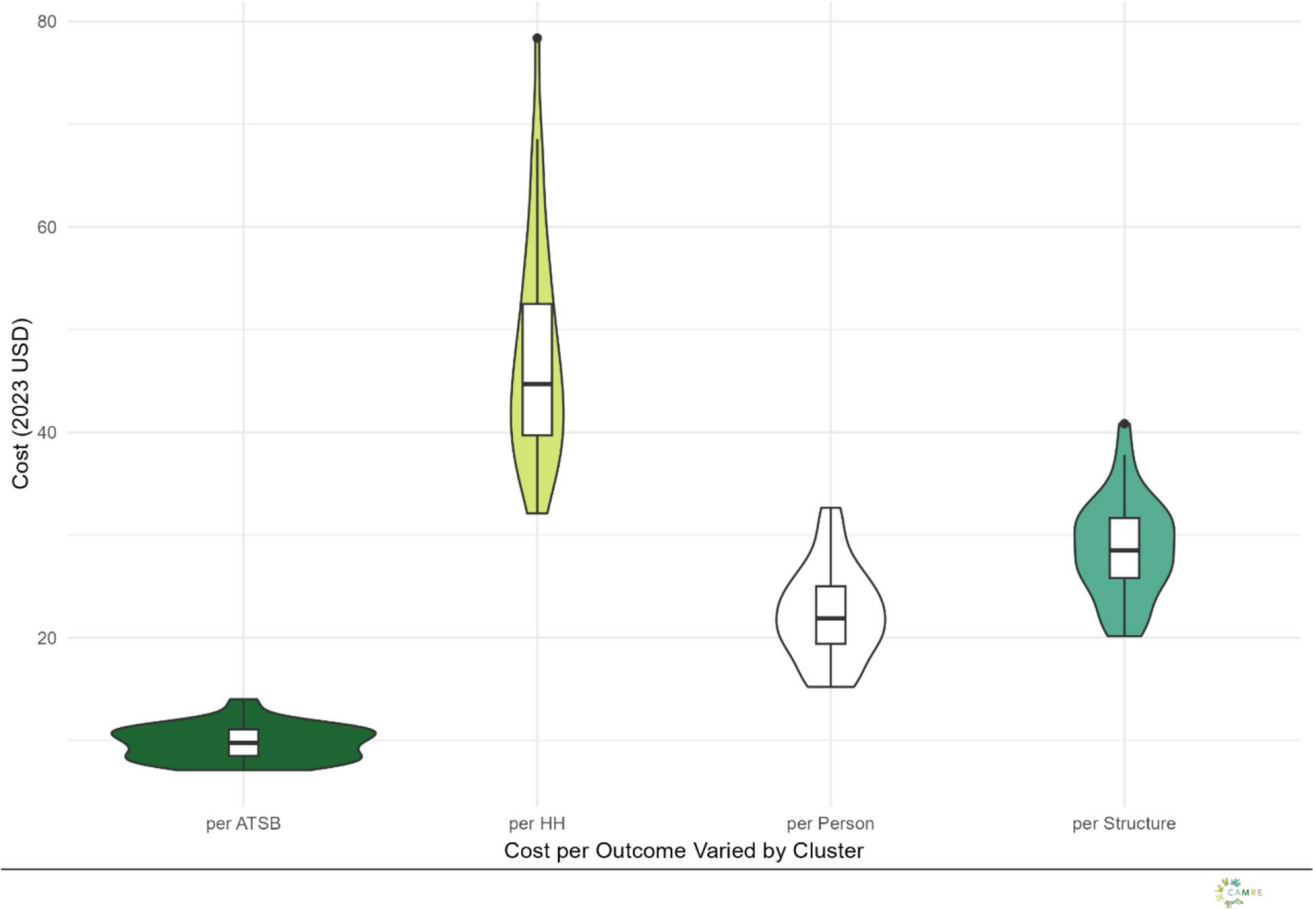
Cluster Level Costs per Outcome: This figure displays the distribution of ATSB intervention costs across clusters in 2023 USD for the base case scenario, presented as cost per ATSB, per household (HH), per person, and per structure. Violin plots illustrate the variability in cluster-level costs, with median values and interquartile ranges overlaid. Costs per household exhibited the greatest variation across clusters, while costs per ATSB were the most consistent

**Fig. 5:**
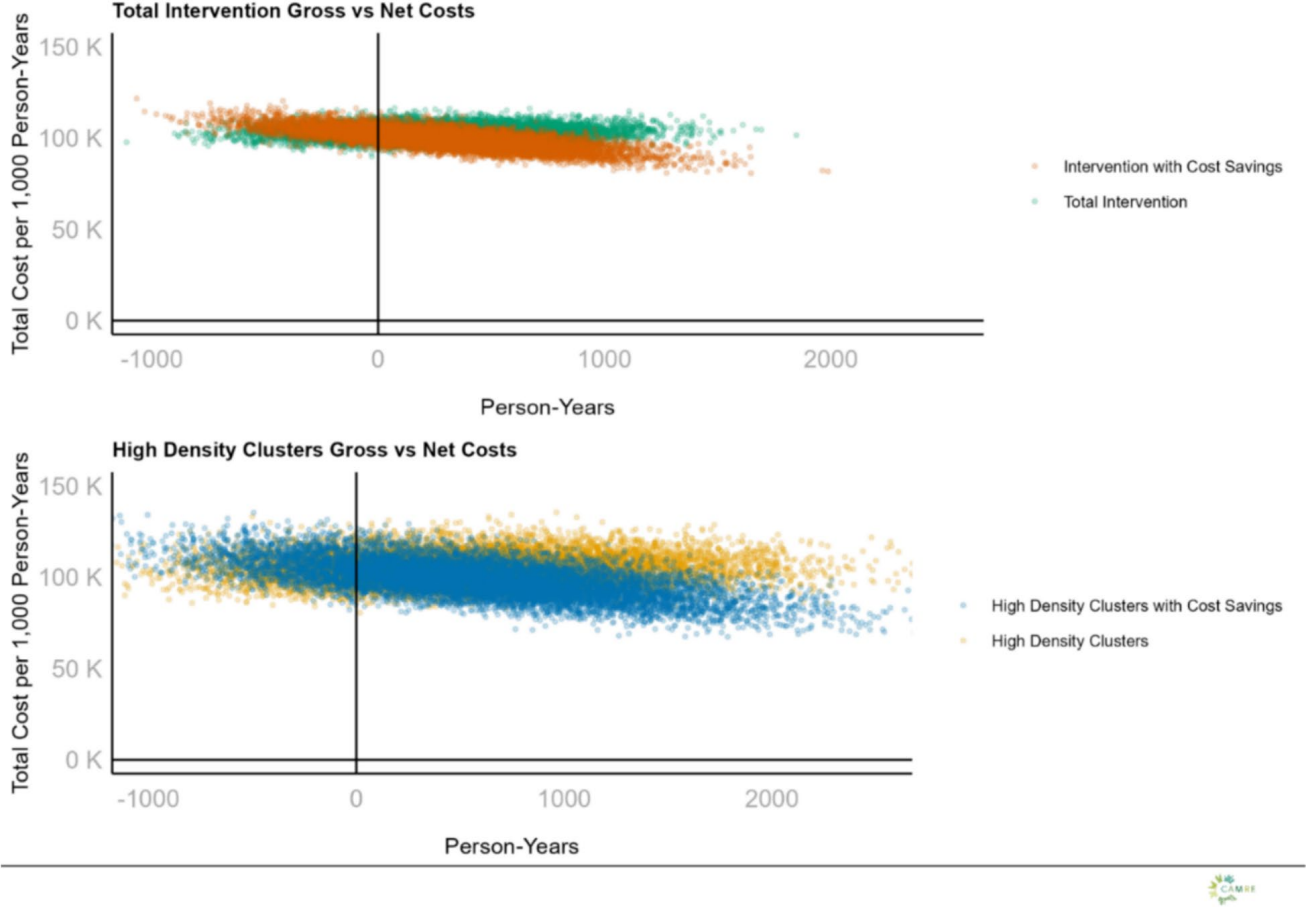
Data Cloud Various Costing Scenarios for ATSB: Each data cloud represents the distribution of results from 10,000 probabilistic iterations for the corresponding scenario. These simulations incorporate parameter uncertainty to reflect variability in cost-effectiveness outcomes

The relationship between the Incremental Cost-Effectiveness Ratio (ICER) and the Incidence Rate Ratio (IRR) was reduced to determine the point at which (ceteris paribus) the intervention would be considered cost-effective. At an IRR of 0.89 (~ 11% reduction in incidence) the intervention would be considered highly cost effective, even if using more strict standards as 0.5 × GDP per capita for Zambia (Fig. 2). Although this IRR point estimate was not statistically significant, it represents the best available central estimate of the intervention effect and is therefore used as the base case in threshold analysis. Threshold analysis, therefore, evaluates whether an effect of this magnitude, if true, would meet cost-effectiveness criteria, accounting for uncertainty through sensitivity analyses (see Figs. 3, 4, 5).

### Probabilistic sensitivity analysis

Probabilistic sensitivity analyses were done utilizing base case scenario assumptions (Gross), base case scenario with cost-savings (Net) and high-density clusters (Gross sub-group) and high-density clusters with cost-savings (Net sub-group) for the two-year intervention period (two transmission seasons). The probability of the intervention being considered cost-effective did not reach 80% at either the 1 × nor 3 × GDP willingness to pay threshold under base case scenario assumptions (Fig. 4). While the high ATSB station and structure density sub-group, both the gross and net cost scenarios closely approached an 80% probability of being considered highly-cost-effective (*e.g.* the ICER had > 80% probability of averting a DALY at a Willingness to Pay Threshold = National GDP per capita), but never met an 80% probability despite any willingness to pay figure (Fig. 3).

## Discussion

This study examined the cost and cost-effectiveness of ATSB as part of a Phase III Cluster Randomized Control Trial to assess the efficacy of ATSB at reducing clinical malaria case incidence in children 1–15 years. The total cost for the intervention over two transmission seasons under base case scenario assumptions was estimated to be USD 1,261,515. The main cost driver was the ATSB stations themselves. Following ATSB station costs, the next largest costs were personnel costs, which arose partly because the extensive monitoring system which was in place during the time in which ATSB stations were hanging in the intervention area study structures to help ensure adequate intervention coverage throughout the trial. Labour costs for ITN and IRS distribution were generally one of the main drivers of cost aside from the costs of the vector control products, which aligns closely with findings from this trial [15, 33]. Routine use of ATSB stations would not likely be monitored as extensively as was done during the course of this trial and as such significant cost reductions would be achieved by scaling back this monitoring system. However, this cost reduction would also likely negatively impact the efficacy/effectiveness of the intervention due to lower coverage and deteriorated condition of some ATSBs. Of note, during monitoring approximately one third of all ATSB station deployments occurred, primarily in response to deteriorated or missing bait stations, and to a lesser extent, the identification of new or missed structures [34]. Thus, removing the monitoring system would remove these costs, but as noted would likely impact negatively impact coverage, ATSB quality, and/or effectiveness. As such it is not possible to speculate on the direction or magnitude of the impact of removing monitoring on the cost-effectiveness or ICER results but only on the overall cost of the intervention. Importantly, ICERs are not the sole criteria for decisions regarding investments in a given intervention; factors such as operational feasibility, equity, health system fit, and stakeholder preferences also play a critical role [35].

In this trial the estimated cost per DALY averted was USD 1,101, which is much higher than USD 48.53, the cost per DALY for ITN from a recent review [31]. Estimates of cost-effectiveness of vector control interventions in the past have largely been made in comparison to no alternative vector control intervention. In this trial the delivery of ATSB stations was conducted against a background of existing universal vector control. As such the estimates cannot be directly compared to a generalized (vs. do-nothing) estimate of ITN or IRS cost-effectiveness. As other vector control tools are rolled out, these may serve as more robust comparisons. For example, in Côte d’Ivoire the addition of lethal house lure incurred an economic cost of USD 249.65 per DALY (after adjusting from 2018 to 2023 figures) [36].

Lethal house lure were a much more effective addition to vector control in Côte d’Ivoire compared to ATSB in Zambia and therefore have a much more favourable cost per DALY(36). In addition, ITN and IRS costs and cost effectiveness will change over time with the introduction of new products to combat insecticide resistance, product price changes, distribution system changes and other factors [33]. Nevertheless, ATSB deployment in the base case scenario were not likely to be considered cost-effective in the western Zambian setting, largely due to the low and uncertain level of effectiveness seen in the trial. Similar findings emerged from the cluster randomized control trial in Mali (IRR = 0.90; 95% CI 0.77—1.05; p = 0.188) [37]. In Kenya, results were likewise not statistically significant and, in fact, suggested a trend in the opposite direction (IRR = 1.11; 95% CI: 0.75 – 1.65; p = 0.598) [38].

Several factors were evaluated to understand variation in and drivers of the ICER for ATSB deployment in these settings. While the cost of ATSBs themselves was the primary cost driver, large-scale manufacturing or changes in the production process could lead to reduced prices for ATSB stations, which therefore could lead to significant improvement in the costs associated with ATSB purchase. The ATSB Sarabi v1.2 is a very new product and products often experience significant declines in production cost as they reach production at scale, competitor products enter the market and manufactures learn to be more efficient with experience in manufacturing processes [31]. Some combination of these factors have led to long term declines in the cost of the manufacture of long-lasting insecticidal net products, and it is reasonable to expect that similar trends could be observed for future ATSB stations if they are found to be effective enough for wide scale deployment [14, 31, 39]. Additionally, significant variation in the cost of deployment was seen between clusters indicating that there may be considerable variation in deployment driven by local conditions including, but not limited to, the built environment.

These factors, including structure density, may lead to changes in both the effectiveness and the cost of deployment for ATSB stations. A post-hoc subgroup analysis of trial data by Ashton et al., suggested that in trial clusters with higher structure density, ATSB station efficacy may have been better than for the trial area as a whole. Consequently, this analysis showed that cost-effectiveness was improved by around 50% for this sub-group. Indicating that local conditions might be used to identify areas where even in the current configuration and with the current product, deployment of ATSB stations in addition to current vector control might meet global definitions for cost-effectiveness. While the ICER estimates, even for the sub-group, remain substantially higher than those for ITN or IRS compared to a do-nothing approach, they show that ATSB deployment may be suitable in some areas even as the current deployment strategy and product might not be an efficient use of resources broadly. Finally, the distribution and monitoring system itself was also costly both because of the strong effort put into monitoring the stations, but also because this monitoring effort led to the consumption of at least 30% more ATSB stations than might have otherwise been used in the course of the trial. In routine use it is almost certain that the intervention would be less costly to deliver than what was seen in this trial, both because the managers of the delivery system for ATSB stations would likely benefit from learning by doing in deployment, as well as from reductions in the intensity of the monitoring system and possibly in some cases from volume discounts on product procurement or from economies of scale or scope that might be captured in the system expansion. While it seems likely that these would lead to reduced costs in routine use, it is almost certain that reduced coverage and deteriorated condition of the ATSBs that would have occurred in the absence of the monitoring system would have a negative impact on overall effectiveness. Given the considerations, the effect of such changes on the cost effectiveness of ATSB stations to prevent malaria remains unclear.

The cost savings estimates from the study show that potential cost savings from the amount of malaria prevented in this trial are likely to be small compared to the cost of delivering the intervention itself. These estimates are also highly speculative since they assume that the cost of treating an uncomplicated malaria case can be fully recovered for each case averted that would have been treated. This is likely to be somewhat true in the case of economic costs, as analysed here, but may be less likely to be fully realized financially since large components of these costs are related to clinician’s time and infrastructure which may not be effectively utilized simply because the malaria burden has been reduced [40].

There are some major limitations to this work. These include, most importantly, the fact that ATSB cost-effectiveness findings in these settings rely on an extrapolation of effects in incidence from a cohort of children less than fifteen years of age to the whole population. Cost-effectiveness under the assumption that ATSB only provided effects on disease incidence in the child population were less attractive. Global assessments of cost-effectiveness here in terms of DALYs averted were also highly sensitive to the choice of case fatality rate which is a generally unknown and likely highly variable metric for malaria. Additionally, the low effect size and significant uncertainty surrounding the effect measure mean that all conclusions are highly uncertain and further research is needed to confirm the efficacy of these or future comparable tools in this setting and especially in settings where the effect size might be maximized.

The distribution strategy chosen for the trial likely had high impacts on both costs and on effect and while some aspects of this result are straightforward to address with sensitivity analysis (e.g. the effects on cost) the impact of choices of distribution strategy on the effectiveness found in the trial cannot be examined without further field studies.

The choice of cost-effectiveness threshold was also a consideration in this analysis. The use of 1–3 × GDP threshold was chosen as this has been used in previous studies on vector control for malaria [13, 14, 39, 41]. However, these thresholds are much more generous than other proposed cost-effectiveness thresholds, which try to take into account the nuance and elasticity of costs and benefits unique to each country. As this analyses showed no cost-effectiveness at the most generous thresholds it stands to reason that they are not cost-effective at the lower proposed thresholds.

The trial compared ATSB to no ATSB in the context of high coverage of standard vector control. Given this backdrop it is not possible to know how effective the ATSB would have been on a background of no standard vector control. As such the ATSB are being asked to prevent the remaining marginal cases left after ITN and/or IRS have already been deployed. It is likely that the efficacy of ATSB as a first intervention would be higher and thus their cost-effectiveness would be better when deployed alone, than what is seen here. Since ITN and/or IRS remain the standard of care for prevention of malaria in settings such as western Zambia, running a trial with no vector control would be unethical, making modelling necessary to estimate ATSB as a first vector control component. As malaria control programmes are likely to face increasingly complex choices in the field of vector control including comparisons of multiple interventions or combinations of interventions, providing more detail on the cost and cost-effectiveness of vector control in different settings and in varied combinations is becoming more and more necessary.

Finally, the trial in western Zambia was conducted in a highly malarious area with significant residual transmission happening even after the deployment of standard vector control interventions. While the reasons for this residual transmission are not currently well understood, one implication of the high malaria incidence and prevalence in the study area is that even small proportional reductions in the incidence of malaria result in substantial numbers of malaria cases averted. This in turn can lead to the intervention appearing to be much more cost-effective than if deployed with similar protective efficacy into areas with lower levels of malaria incidence. When considering the deployment of ATSB stations in other areas, consideration of malaria transmission and the potential to avert cases is an important component of the translation of these results.

## Conclusions

ATSBs Sarabi v1.2 deployed two per eligible structure during the transmission season are not expected to be a cost-effective tool for widespread use as a malaria prevention strategy in Zambia. There are indications that they may be more cost-effective if deployed in areas with higher structure densities, but they were not based on statistically significant efficacy values and still fell below the ideal cost-effectiveness likelihood threshold of 80%. Including cost-savings from averted malaria cases to the provider (health system) did not affect this conclusion. Stronger evidence of effectiveness and costs under alternative distribution strategies will be needed before any large-scale role for ATSB can be justified.

## Supplementary Information

Additional file 1.

## Data Availability

De-identified data are available from the corresponding author on reasonable request. Following publication of forthcoming secondary analyses of trial data, the deidentified trial dataset will be posted on a public repository.

## Abbreviations

ATSB: Attractive Targeted Sugar Bait
BLS: Bureau of Labor Statistics
CFR: Case Fatality Rate
CHW: Community Health Worker
cRCT: Cluster Randomized Controlled Trial
CEAC: Cost-Effectiveness Acceptability Curve
CHEERS: Consolidated Health Economic Evaluation Reporting Standards
DALY: Disability-Adjusted Life Year
DSA: Daily Subsistence Allowance
GBD: Global Burden of Disease
GDP: Gross Domestic Product
GLM: Generalized Linear Model
HH: Household
ICER: Incremental Cost-Effectiveness Ratio
IRS: Indoor Residual Spraying
IRR: Incidence Rate Ratio
ITT: Intent-to-Treat
ITN: Insecticide-Treated Net
MOH: Ministry of Health
NMEC: National Malaria Elimination Centre
PATH: Program for Appropriate Technology in Health
PSA: Probabilistic Sensitivity Analysis
RDT: Rapid Diagnostic Test
SBCC: Social and Behaviour Change Communication
USD: United States Dollar
YLD: Years Lived with Disability
YLL: Years of Life Lost
ZMW: Zambian Kwacha
