# Supplementary material for: Cost and cost-effectiveness of attractive targeted sugar baits (ATSB) in the context of a phase III cluster randomized control trial in Western Province, Zambia
Source: Malar J. 2025 Dec 19;25:54. doi: 10.1186/s12936-025-05716-9 (PMC12831337; doi:10.1186/s12936-025-05716-9)
Supplement: Supplementary file 1 — Additional file 1. [file 12936_2025_5716_MOESM1_ESM.docx]

**Supplementary Appendices**

**Intervention Description**

ATSB stations were delivered to Lusaka by air and cleared customs with no demurrage fee in Year 1. In Year 2 ATSB stations were shipped to Dar es Salaam and transported by semi-truck to Lusaka, with no fee upon entry. They were then brought to Kaoma, Western Province and stored alongside other ATSB distribution supplies in a climate-controlled storage unit.

***Sensitization (Pre-Deployment)***

Before ATSB stations were distributed in October 2021 and October 2022, community sensitization in the 35 intervention clusters took place. Community sensitization then continued at a smaller scale throughout the course of the intervention.

Seventy-two ATSB Community Health Workers (CHWs) (2-4 per cluster) were hired from October 2021 through June 2022 in year 1, and 80 ATSB CHWs were hired for October 2022 and December 2022-June 2023 in year 2, in intervention clusters. Responsibilities for these CHWs included sensitization and responding to community concerns during installation, monitoring, and hang-down activities. The primary sensitization activities included meetings with local leaders and door-to-door sensitization with community members. Due to ongoing COVID restrictions in the first year, CHWs were not able to conduct large community meetings. However, for year-two large meetings did take place. Small incentives for local leaders, such as cooking oil, chicken, and maize flour were provided.

Dedicated on-site briefings at 28 community health facilities were conducted in early October 2021 for year 1 only. Key personnel in these meetings were the nurses in charge, and outpatient staff. Health facility staff participated in a 1-day training in Kaoma in January 2022, in response to some isolated challenges with ATSB station removals and arising questions/rumors about ATSBs. In November 2022, for year 2 of the intervention, health facility staff instead traveled to Kaoma for a centralized training.

During both intervention years, from December 2021 to June 2022, and again between December 2022 and June 2023, the ATSB team from Kaoma held meetings when necessary, in response to ATSB removal with the CHWs. Educational materials describing the purpose of ATSB stations were distributed, and airtime and advertising on a radio talk-show and radio jingles were also used to increase acceptance and awareness of ATSBs throughout the duration of the trial.

***Installation***

Oversight of the ATSB deployment, including installation, monitoring, and hang-down was conducted by community-based ATSB monitors. Installation training for ATSB Monitors took place in late October in 2021 and again in October 2022 in Kaoma over a period of three or two days, respectively. Per diem was provided for the duration of the training.

ATSB station installation took place between November 1-17, 2021 in year 1 and October 31-November 12, 2022 in year 2. ATSB installation included three days of ‘mop-up’ activities to confirm sufficient coverage across intervention clusters. Six ATSB staff were responsible for overseeing ATSB station installation in the 35 clusters. Each officer was assigned 5-6 clusters to support during installation and had a dedicated car-hire vehicle for the span of installation.

In Year 1, 280 surge-support installers (eight installers per cluster) were provided with a half day training on-site in their respective clusters. In Year 2, 177 surge-support installers were recruited and ATSB monitors, CHWs, and surge support installers made up the installation teams in each cluster.

During installation in Year 1, teams worked in pairs with one individual conducting data entry, determining structure eligibility, and obtaining consent, while the second individual prepared and installed the ATSB station. Five phones were dedicated to each cluster and managed by the CHWs to record the installation of ATSB stations. Three of those phones per cluster were collected back at the end of the deployment period, with the remaining two phones left with ATSB monitors to use during the upcoming monitoring activities. Each phone was provided airtime credit to sync installation data.

Year 2 installation differed in that installation teams worked in teams of 3, with the ATSB monitor conducting data entry and determining structure eligibility, the CHW providing household consent and sanitization, and a surge support individual physically preparing and installing the ATSB station on the structure. In year 2, only the 87 ATSB Monitors were given phones, which were provided with airtime to sync installation data.

Supplies for ATSB station installation included ATSB stations, bamboo sticks, wire, wire cutters or pliers, nails, hammers, gloves, and biohazard bags. Pens, notepads, SBCC materials and maps were also provided. Each of the ATSB Monitors received a bicycle, phone, and battery pack (power pack chargers). One ATSB Monitor per cluster was also given lockable storage boxes that could fit about 60 - 100 new ATSB stations and biohazard waste, so that all were kept in a safe location inside of the ATSB Monitors home. There was no additional financial payment provided to ATSB monitors for the use of storage space inside their homes.

ATSB Monitors determined which structures were eligible for hanging ATSB using the guidelines outlined in the trial protocol and training sessions (20). All eligible structures at consenting households had two ATSB stations installed.

***Monitoring***

Two full-time staff, known as ATSB Officers, were responsible for overseeing ATSB Monitors in all 35 clusters (17 and 18 clusters respectively for each ATSB Officer). During the monitoring period, two cars were dedicated to the officers for monthly cluster visits and supervision in both years. Some clusters received more frequent visits to respond to community concerns, data queries/concerns, or other issues. Two ATSB motorbike drivers were also on-hand to deliver additional supplies between these monthly visits if necessary.

Seventy-nine ATSB Monitors in year 1 and eighty-seven ATSB Monitors in year 2 (two per cluster except in seven clusters where the extended geographic area required additional personnel) were hired with the responsibility of monitoring and replacing ATSB stations during the trial **(**74 ATSB monitors remained the same as the ATSB monitors trained prior to deployment, 5 additional monitors were brought on after January 2022 in year 1). ATSB monitors received a two-day training on ATSB monitoring and replacement expectations before the start of monitoring activities. Monitors worked 15 days per month and were expected to monitor each ATSB station at least once every two months, replace damaged ATSB stations, and install ATSB stations on newly identified or built structures. Additional visits to structures with ATSB station damage were sometimes required in between routine monitoring visits. ATSB Monitors were paid USD 4.95 per day for a maximum of USD 74.26 per month (this was regardless of any damage visits made outside of the routine visit schedule).

ATSB Monitors continued to utilize the same phones as in installation and received monthly airtime payments. ATSB Monitors kept an adequate stock of ATSB stations, other replacement materials, and waste in the lockboxes in their clusters. The ATSB Officer would collect any waste from discarded/replaced ATSB stations and bring it back to the Kaoma storage room in biohazard bags. ATSB waste was then kept in drums until they could be transported to Lusaka for incineration. Three trips to transport waste from Kaoma to Lusaka during the monitoring took place in both years.

***Hang-Down***

Community sensitization specific to hang-down took place from May 22, 2022, to May 31, 2022 and from May 29, 2023 to June 9, 2023 in the second year. It was conducted by three ATSB Community Engagement Officers. This sensitization included community meetings and meetings with chiefs and other local leaders. Local chiefs were again provided with small incentives. Additionally, community contact cards with information on the hang-down process were printed and given to every household at the time of ATSB station removal.

Five full-time ATSB Staff were responsible for overseeing ATSB station hang-down in the thirty-five clusters. Each officer was assigned 6 clusters to support during hang-down and had a dedicated vehicle for the duration of the hang-down process. Three full-time staff from the Kaoma operations team were responsible for receiving and handling waste at the storage site in Kaoma.

In each cluster, there were 2-4 hang-down teams. Each hang-down team consisted of one ATSB Monitor, one CHW and one surge support person; teams were responsible for data entry and physically removing the ATSB stations and associated hanging materials from the structure and packaging it for disposal. Everyone was paid a daily rate for a maximum of 12 working days.

Half day hang-down trainings for the ATSB Monitors, CHWs and surge support took place from June 1 - June 10, 2022 in year 1. A full-day training for hang down was conducted in Year 2 for the same staff. A per diem to cover daily subsistence allowance (DSA) during the training period was given. PATH also provided stationary, lunch and water in year 2. Training included data entry training, key messages for the community, safety, and waste handling. All clusters were supplied with extra biohazard bags, gloves, hammers and pliers, and bikes. All supplies necessary for the hang-down process were brought to their dedicated locations before the start of the hang-down process on June 13 - 14, 2022 for year 1 and June 13-15, 2023, for year 2.

Hang-down took place daily from June 15 – July 1, 2022, and from June 15-29, 2023 in for years 1 and 2, respectively. This included a few days of additional ‘mop-up’ time to allow for the location of missed ATSB within study clusters.

All the biohazard materials were loaded into drums and transported by a Ministry of Health (MOH) flatbed semitruck to Lusaka. The materials were then incinerated by a third-party vendor in Lusaka using high temperature incineration.
